# Endophytic commensal bacteria capitalize on the AvrPto-FER pathway to enhance proliferation during early stages of pathogen invasion

**DOI:** 10.1093/ismejo/wraf145

**Published:** 2025-07-09

**Authors:** Yi Zhang, Dan Hu, Hong-xia Sun, Jia Chen, Jia-hao Yang, Xin-mei Li, Xiu-shan Li, Yan Chen, Feng Yu

**Affiliations:** State Key Laboratory of Chemo/Biosensing and Chemometrics, College of Biology, Longping Agricultural College, Hunan University, Changsha 410082, China; Yuelushan Laboratory, Changsha 410128, China; Hunan Institute of Microbiology, Changsha 410009, China; Hunan Institute of Microbiology, Changsha 410009, China; State Key Laboratory of Chemo/Biosensing and Chemometrics, College of Biology, Longping Agricultural College, Hunan University, Changsha 410082, China; Yuelushan Laboratory, Changsha 410128, China; State Key Laboratory of Chemo/Biosensing and Chemometrics, College of Biology, Longping Agricultural College, Hunan University, Changsha 410082, China; Yuelushan Laboratory, Changsha 410128, China; State Key Laboratory of Chemo/Biosensing and Chemometrics, College of Biology, Longping Agricultural College, Hunan University, Changsha 410082, China; Yuelushan Laboratory, Changsha 410128, China; State Key Laboratory of Chemo/Biosensing and Chemometrics, College of Biology, Longping Agricultural College, Hunan University, Changsha 410082, China; Yuelushan Laboratory, Changsha 410128, China; State Key Laboratory of Chemo/Biosensing and Chemometrics, College of Biology, Longping Agricultural College, Hunan University, Changsha 410082, China; Yuelushan Laboratory, Changsha 410128, China; State Key Laboratory of Chemo/Biosensing and Chemometrics, College of Biology, Longping Agricultural College, Hunan University, Changsha 410082, China; Yuelushan Laboratory, Changsha 410128, China; State Key Laboratory of Chemo/Biosensing and Chemometrics, College of Biology, Longping Agricultural College, Hunan University, Changsha 410082, China; Yuelushan Laboratory, Changsha 410128, China

**Keywords:** endosphere, microbial community, commensal bacteria, receptor kinase, immunity

## Abstract

Leaves typically harbor a community of pre-existing beneficial and commensal bacteria that contribute to plant health. When pathogens invade, plants employ a series of strategies to response to the invasion, including the modulation of the microbial community structure. However, it remains unclear how commensal bacteria respond to pathogen at the early stage, and whether this response is specifically regulated. Here, we show that infection of *Arabidopsis thaliana* leaves by the pathogen *Pseudomonas syringae* pv. *tomato* DC3000 leads to a significant increase in the population of commensal bacteria, characterized by enrichment of Gammaproteobacteria and Alphaproteobacteria, alongside a reduction in Firmicutes and Betaproteobacteria. This cascade of events specifically occurs because AvrPto, an effector secreted by *Pst* DC3000, targets and inhibits the host receptor kinase FER, resulting in suppression of FER-mediated pattern-triggered immunity via the previously identified RIPK–RBOHD module. This specific suppression via FER pathway creates a condition that facilitates rapid proliferation of pre-existing commensal bacteria during early pathogen invasion. Our work provides a paradigm for the study of the interaction and ecological generality between commensal bacteria and pathogens with spatiotemporal patterns.

## Introduction

The plant microbiome, consisting of the community of microorganisms living in and on plants, influences plant growth, health, and adaptation to environmental conditions [1]. The population of pre-existing commensal and beneficial bacteria in the microbiome is kept within a reasonable range during most of the plant life cycle [2]. However, under a specific condition or at a certain time, pathogenic bacteria attack leaves and rapidly proliferate, causing significant damage to plants [3]. Uncontrolled proliferation of both commensal and pathogenic bacteria negatively affects plant health [2]. For example, high population densities of the non-pathogenic bacterium *Williamsia* sp. Leaf354 induce disease phenotypes in Arabidopsis (*Arabidopsis thaliana*) leaves. Thus, the roles of commensal or pathogenic bacteria are not always invariable during evolution [4], which complicates the interactions between beneficial and harmful bacteria and their transformation.

Commensal bacteria respond to the invasion of the host by inducing plant immune responses [5], competitive exclusion [6], and antibiosis [7]. Pathogens and commensal bacteria interact and compete for access to colonization sites and resources in the phyllosphere. For example, competition between *Sphingomonas* strains and the leaf pathogen *P. syringae* for host-derived carbon sources limits pathogen growth in Arabidopsis [8]. The commensal strain *Pseudomonas putida* utilizes the type VI bacterial secretion system to inject toxic effector proteins into target cells, killing the plant pathogenic bacterium *Xanthomonas campestris* and protecting plant leaves from this pathogen [9]. Recently, a small molecule, 2,4-di-tert-butylphenol, secreted by an *Aspergillu*s-enriched microbiota, was reported to effectively protect crop leaves against *Rhizoctonia solani* infection [10]. Even if the pathogen evades suppression by the commensal bacteria, quorum quenching activities can interfere with the expression of virulence genes in the pathogen by degrading quorum-sensing signals, thereby suppressing disease progression [11]. However, all of the above responses and mechanisms are relatively downstream events that occur following the detection of pathogen invasion by commensal bacteria. It remains unclear how commensal bacteria respond to pathogen invasion at the early stage, and whether this response is specifically regulated.

Many gram-negative pathogenic bacteria utilize the type III secretion system to deliver effector proteins directly into eukaryotic cells, allowing cellular pathways in the host to be manipulated for the benefit of the pathogens [12]. Loss of these effector proteins result in reduced or complete loss of pathogenicity. The deletion of 28 highly expressed effector genes in model pathogen *P. syringae* pv. *tomato* (*Pst*) DC3000 (resulting in the DC3000^*Δ*28E^ mutant) led to a significantly reduced ability to induce cell death in plants compared to wild-type *Pst* DC3000 [13]. Effectors are injected into plant cells to manipulate components of the immune system, including pattern-triggered immunity (PTI) [14] and effector-triggered immunity (ETI) [15, 16]. For instance, the effector AvrPto targets pattern recognition receptors (PRRs) such as Flagellin-sensitive 2 (FLS2) and EF-Tu receptor (EFR), thereby suppressing plant immunity and promoting the growth of pathogenic bacteria within the plant [17, 18]. However, the early dynamic changes of commensal bacteria during pathogen proliferation in the process of plant immune responses remain unclear.

To elucidate how commensal bacteria respond to pathogen invasion during the early stages, we isolated leaf endophytic bacteria and analyzed their dynamics in response to pathogens in the *FER* mutant, which was previously shown to influence the phyllosphere (not endosphere) microbiome [19]. Furthermore, genetic and biochemical experiments demonstrated that the effector AvrPto from *Pst* DC3000 inhibits the receptor kinase FER, thereby dampening RIPK–ROBHD–mediated immunity. This suppression allows pre-existing commensal bacteria to rapidly proliferate in response to pathogens. Our findings reveal that leaf endophytic commensal bacteria respond to pathogen invasion at an early stage via a relatively specific immune pathway regulated by the receptor kinase FER. These insights shed light on the complex interactions among plants, pathogens, and commensal microbes mediated by effector-driven immune manipulation, paving the way for microbiome-based strategies to control plant diseases.

## Materials and methods

### Plant materials and growth conditions

The Arabidopsis (*A. thaliana*) accession Col-0 was used as the wild type. The *fer-4* [20], *fls2* [17], *bak1–4* [21], *fec* [22], *ripk* [23], *eds1–22* [24], *fer-4*/FER^P740A^ [25], *fer-4*/FER^WT^ [25] and *rbohD* [19] mutants were described previously. To generate *pACTIN2::avrPto* transgenic plants, the full-length *avrPto* gene from *Pst* DC3000 was cloned into the plant expression vector pDT1 [26] under the control of the *ACTIN2* promoter. All seeds were surface-sterilized with 75% ethanol for 5 min and 10% NaClO (v*/*v) for 5 min, washed at least three times with sterile water, vernalized at 4°C for 3 days in the dark, and sown on 1/2-strength Murashige and Skoog (MS) medium with 0.8% (w*/*v) sucrose and 1% phytagel for subsequent analysis. The plants were maintained under a 12-h photoperiod at 22°C.

For plants grown in soil, vernalized seeds were transplanted into pots filled with an equal volume of peat soil (Pindstrup, Denmark) and vermiculite. The pots were placed in a greenhouse maintained at 22°C with 55% relative humidity and a light intensity of 100 μE m^−2^ s^−1^ under a 12-h photoperiod.

For seedlings grown on plates, seeds were germinated for 3 days and transferred to deep culture dishes containing 1/2× MS without sucrose, solidified with 0.3% Phytagel (Sigma-Aldrich, USA). The plants were grown in a growth chamber at 22°C with a 10-h photoperiod. Two-week-old seedlings were sprayed with bacterial suspension and harvested to count bacteria.

### Bacterial isolation and synthetic communities of leaf endophytic bacteria

Leaves were harvested from 4-week-old soil-grown Col-0 and *fer-4* plants, with eight leaves collected from four plants per genotype (two from each plant). The leaves were surface-sterilized by immersing them in 75% ethanol for 1 min, followed by two rinses in sterile water. The leaves were ground in sterile water, and bacterial suspensions were diluted and plated on R2A plates, which were kept at 22°C for 4 days (Fig. S1A). Approximately 40 colonies per genotype were picked, and the 16S rRNA genes of the colonies were amplified using the primer pair 27F/1492R (see Supplementary Table 1) [27]. The resulting amplicons were purified and sequenced at Sangon Biotech Co., Ltd. (Shanghai, China). After removing duplicate sequences, the sequences were aligned against the NCBI BLAST database. Three endophytic bacterial strains (*Bacillus* sp. C3, *Micrococcus* sp. C5, and *Blastomonas* sp. C10) from Col-0 leaves were used to construct commensal bacterial communities. All 11 endophytic strains from Col-0 leaves, along with 11 selected endophytic strains from *fer-4* leaves, constituted SynCom^Col-0^ and SynCom*^fer-4^*.

To examine the effects of the SynCom on the phenotypes of Col-0 leaves, individual bacterial strains were cultured at 28°C and collected by centrifugation. The strains were suspended by sterile H_2_O, and the bacterial suspensions were adjusted to the same OD_600_. Equal volumes of each strain were pooled and diluted to a final OD_600_ of 0.01. The SynCom bacteria were injected into the leaves of 4-week-old Col-0 plants with a needleless syringe. The plants were continuously cultivated under normal greenhouse conditions, and leaf phenotypes were observed after 5 days of growth.

For experiments conducted on agar plates, ~1 ml of SynCom^Col-0^ or SynCom*^fer-4^* was sprayed onto 2-week-old germ-free Col-0 plants. The phenotypes of leaves were observed after 3 days, and the populations of leaf endophytic bacteria were counted.

### Bacterial infection and quantification of endophytic leaf bacterial microbiota

For the bacterial infection assays on germ-free plants in plates, seedlings were grown on 1/2× MS medium without sucrose in culture plates for 2 weeks before being spray-inoculated with bacteria. The commensal bacteria were cultured overnight in R2B medium at 28°C and resuspended in sterile water containing 0.025% Silwet L-77 (Coolaber, China), and the concentration of the strains was adjusted to an optical density of 10^6^ colony-forming units (CFU) ml^−1^. Approximately 1 ml of bacterial suspension was sprayed onto a culture plate containing 2-week-old plants and incubated for 2 min at room temperature. After removing the residual bacterial suspension by decantation, the plates were incubated in a growth chamber with a 10-h photoperiod at 22°C. To evaluate the effect of the kinase inhibitors reversine and lavendustin A (MedChemExpress, China) on the response of the commensal community to pathogens, 5 μM of each inhibitor was sprayed onto the plates on the first day. To assess the impact of pathogens on the commensal community, the seedlings on the plates were sprayed with pathogens on the third day of culture. The pathogens *P. syringae* pathovar (pv.) *maculicola* ES4326 (*Psm* ES4326) [28], *Pst* DC3000, and *Pst* DC3000 mutants DC3000^*Δ*28E^ [29], D28E(*avrPto*) [29], and DC3000(*ΔavrPto*) [30] were previously described. The leaves were collected at the indicated time points for bacterial counts.

To quantify the culturable endophytic bacterial community, leaves were weighed, surface-sterilized in 75% ethanol for 1 min, and rinsed twice in sterile water. Leaves were ground in 1 ml sterile 10 mM MgCl_2_ buffer. A serial dilution was made and plated on R2A plates (100 mm × 100 mm). CFUs on R2A plates were counted after incubation at room temperature for 3 days, and the counts were normalized to the fresh weight of the leaves. To calculate the number of commensal bacteria in the absence of pathogens, the bacteria on the R2A plates were counted, as they all represented commensal bacteria. To determine the number of commensal bacteria in the presence of pathogens, the bacteria on R2A medium were counted, and the number of pathogens on R2A medium supplemented with 100 mg ml^−1^ streptomycin for *Psm* ES4326, 25 mg ml^−1^ rifampicin for *Pst* DC3000 and its mutants was subtracted.

Using real-time polymerase chain reaction (PCR) to analyze the populations of the commensal bacterial community by treated with non-pathogen *Nitrobacter* sp. C2. The primers were designed based on the specific sequence of *Bacillus* sp. C3, *Micrococcus* sp. C5, and *Blastomonas* sp. C10, which are listed in Supplementary Table 1. The real-time PCR assays were performed using a SupRealQ Ultra Hunter SYBR qPCR Master Mix (Vazyme, China). Standard curves were generated using 10-fold serial dilutions of a plasmid containing the V4 region of the three strains. Each DNA sample was analyzed in three replicates. After calculating the copy number of each target fragment from the standard curves, the results were expressed as log10 values (copies/g leaf).

To quantify the bacterial population of soil-grown Arabidopsis leaves, two leaves each were collected from Col-0 or *fer-4* plants. The endophytic bacteria were counted using the same method used to assess the endophytic bacterial community. Total leaf bacteria were counted following the same protocol except without surface sterilization. A serial dilution was made and plated onto R2A plates.

### Sequencing of 16S rRNA gene amplicons

To analyze the effect of the pathogen *Pst* DC3000 on the endophytic bacterial community of soil-grown Arabidopsis, a low concentration of *Pst* DC3000 (10^5^ CFU ml^−1^) suspended in sterile H_2_O was injected into the leaves of 4-week-old Arabidopsis plants using a needleless syringe. The leaves were collected 24 h later, surface-sterilized with 75% ethanol for 1 min, and rinsed twice with sterile ddH_2_O. The leaf surfaces were blotted dry with autoclaved blotting paper. Two leaves from the same Col-0 plant and four leaves from the same *fer-4* plant were separately collected in one tube as a single biological replicate. To analyze the total bacterial community, leaves were collected from the plants and immediately placed in a tube, snap-frozen in liquid N_2_, and stored at −80°C.

Total DNA was extracted from leaf samples using a MagPure Soil DNA LQ Kit (Magen, China) according to the manufacturer’s instructions. PCR amplification of the v5/v6 region of the 16S rRNA gene was performed using primers 799F/1193R (see Supplementary Table 1) and Tks Gflex DNA Polymerase (Takara, Japan). The amplicons were assessed for quality using gel electrophoresis and purified with AMPure XP beads (Agencourt, USA). Amplicon concentrations were quantified using a Qubit dsDNA assay kit (Life Technologies, USA) and normalized to 3–8 ng μl^−1^. The purified amplicons were pooled for subsequent sequencing. The library was sequenced on NovaSeq platform (Illumina) at OE Biotech Co., Ltd. (China).

### Processing of 16S rRNA gene amplicon sequencing data

Raw 16S rRNA gene amplicon sequencing data in FASTQ format were processed using QIIME2 [31] with default parameters. The paired-end reads were preprocessed using Cutadapt software [32] to detect and cut off the adapter. The sequences were filtered, denoized, and created an amplicon sequence variants (ASV) table by DADA2 [33]. Taxonomic assignment of each ASV was performed using a Naive Bayes classifier [34] pre-trained on the SILVA 16S rRNA gene reference database [35,36] (release 138; https://www.arb-silva.de/). The sequences that were taxonomically assigned to archaea, chloroplasts, mitochondria, and injected *P. syringae* were removed. Diversity analysis was performed using QIIME2.

### Structural analyses of protein complexes

The complexes of 28 effectors from *Pst* DC3000 with FER were modeled using ColabFold [37], which provides a measure of predicted model confidence as a per-residue predicted local distance difference test (pLDDT) score. The structures were analyzed and displayed using PyMOL.

### Luciferase complementation assay

The luciferase complementation assays were performed as previously described [38]. The cDNA of *FER* was amplified and cloned into 35S-pCAMBIA1300-Nluc [39], and the *avrPto* gene was amplified from *Pst* DC3000 using the primers listed in Supplementary Table 1 and cloned into 35S-pCAMBIA1300-Cluc. *Agrobacterium tumefaciens* strain GV3101 carrying these plasmids were co-infiltrated into *Nicotiana benthamiana* leaves. Plants were placed at 23°C for 48 h, covered with plastic bags. Luminescence images were captured using a CCD imaging system after applying the reaction substrate D-luciferin (Biovision, China).

### Yeast two-hybrid assay

Yeast two-hybrid assays were performed as described previously [40]. The coding sequence of FER was cloned into pGBKT7 (BD domain), and the coding sequences of 12 effectors from *Pst* DC3000 (pLDDT >70) were cloned into pGADT7 (AD domain). The amplified primers are listed in Supplementary Table 1. pGADT7 and pGBKT7 served as a negative control. Co-transformed yeast (*Saccharomyces cerevisiae*) strain AH109 was screened on synthetic dextrose medium lacking leucine and tryptophan (SD/-Leu-Trp). The single yeast colonies were serially diluted onto SD/-Leu-Trp and SD/-Leu-Trp-His to observe yeast cell growth.

### Protein expression and purification and GST pull-down assay

The coding sequence of AvrPto was amplified from *Pst* DC3000 genomic DNA with pairs of gene-specific primers by PCR and cloned into pGEX4T-1 (GST-AvrPto). The GST-AvrPto proteins were expressed in *Escherichia coli* BL21 (DE3) and purified using Pierce Glutathione Agarose according to the manufacturer’s instructions (Thermo Fisher Scientific, USA). His-tagged FER-KD was obtained as previously described [41]. See Supplementary Table 1 for the primer sequences used.

Recombinant FER-KD-His (2 μg) was incubated overnight at 4°C with GST beads coupled with 2 μg GST-AvrPto in binding buffer (20 mM HEPES [pH 7.5], 40 mM KCl, 5 mM MgCl_2_) [23]. The beads were washed three times with Tris-buffered saline (50 mM Tris [pH 7.5], 150 mM NaCl), and the proteins were analyzed by SDS-PAGE, followed by immunoblotting with anti-His (Abcam, USA) and anti-GST (ABclonal, USA) antibodies.

### Co-immunoprecipitation assay

Co-immunoprecipitation (Co-IP) assays were performed as previously described with some modifications [42]. Seven-day-old *pACTIN2::avrPto* (~0.5 g) seedlings were ground to a fine powder in liquid nitrogen, solubilized with 500 μl TBST buffer (50 mM Tris–HCl [pH 7.5], 150 mM NaCl, 5 mM MgCl_2_, 1 mM ethylene diamine tetraacetic acid [EDTA], 1% Triton X-100) containing 1× protease inhibitor cocktail (Thermo Fisher Scientific, USA) and 1× phosphatase inhibitor (Thermo Fisher Scientific, USA), and incubated for 1 h at 4°C. The extracts were centrifuged at 12 000 g at 4°C for 10 min, and 500 μl of the supernatant was collected and incubated with 20 μl anti-myc magnetic beads (NuoyiBio, China) overnight at 4°C; 100 μl of the supernatant was used as input. The beads were washed three times with washing buffer (50 mM Tris–HCl [pH 7.5], 150 mM NaCl, 0.1% Triton X-100) containing 1× protease inhibitor cocktail and eluted with elution buffer (0.2 M glycine, 1% Triton X-100, 1 mM EDTA, pH 7.5). The proteins were separated by 10% SDS-PAGE and analyzed by immunoblotting with anti-FER (6 × His-FER-CD was purified and used as an antigen to produce a polyclonal antibody in mouse) [23] and anti-myc (Cell Signaling Technology, China) antibodies.

### *In vitro* phosphorylation assay

The *in vitro* phosphorylation assays were performed as described as previously [23]. Buffer containing 50 mM HEPES (pH 7.5), 5 mM MgCl_2_, and 10 mM ATP was used for FER-KD autophosphorylation and phosphorylation. All reactions were incubated at 25°C for 30 min and terminated by adding 4× SDS loading buffer. The proteins were fractionated by sodium dodecyl sulfate-polyacrylamide gel electrophoresis (SDS-PAGE), and the phosphorylated proteins were analyzed by immunoblotting with anti-phosphoserine (anti-Ser) and anti-phosphothreonine (anti-Thr) antibodies (Abcam, USA). To determine the effect of AvrPto on FER-KD kinase activity, the phosphorylation assay was carried out in the presence of different concentrations of AvrPto (2 to 20 μM).

### *In vivo* phosphorylation assay

To examine the effects of *Pst* DC3000 and the mutants on the phosphorylation of FER *in vivo*, the bacterial strains were cultured overnight in 28°C, collected, and washed twice with sterile water. The bacterial suspension was adjusted to OD_600_ = 0.1 and Col-0 seedlings were incubated in the bacterial suspension for 8 h. The seedlings were ground and subjected to protein extraction in extraction buffer (20 mM HEPES pH 7.5, 150 mM NaCl, 10% [v/v] glycerol, 1% [v/v] Triton X-100, 5 mM EDTA, 10 mM DTT, 1 mM phenylmethylsulfonyl fluoride (PMSF), and halt protease inhibitor cocktail [Thermo Fisher Scientific, USA]) for 1 h. The samples were centrifuged at 12 000 g for 10 min at 4°C, and the supernatants were collected. The samples were run on a 10% SDS-PAGE gel and analyzed by immunoblotting with anti-FER and anti-FER^pY648^ antibodies (ABclonal, China).

To examine the effects of AvrPto on FER phosphorylation in *pACTIN2::avrPto* plants, phosphorylated bands in 10-day-old *pACTIN2::avrPto* seedlings were analyzed *in vivo.* As a positive control, 200 nM RALF1 was incubated seedlings in seedlings. Total proteins were extracted from the seedlings using extraction buffer. The samples were analyzed by SDS-PAGE and immunoblotting with anti-FER^pY648^ (ABclonal, China), anti-FER^pS701^(ABclonal, China), anti-FER, and anti-myc (Cell Signaling Technology, China) antibodies.

### Statistical analysis

Raw data for total and endophytic leaf bacteria, and real-time PCR were processed using Microsoft Excel software (Microsoft, USA). GraphPad Prism 10.0 (GraphPad, USA) was employed to generate line charts and stack plots, as well as to perform statistical analysis. Student’s t-test or two-way ANOVA with Tukey’s HSD (*P* < 0.05) was applied to evaluate differences between treatments, as described in figure legends. Each measurement in this study was based on three biological replicates, with at least three technical replicates per biological sample.

## Results

### *Pst* DC3000 invasion leads to rapidly increased commensal bacteria levels in Arabidopsis leaves

Under normal conditions, commensal bacteria are the predominant members of the leaf microbiota [2]. To explore the early steps in the response of commensal bacteria to pathogen invasion, we first obtained commensal bacteria by isolating endophytes from the leaves of wild-type Arabidopsis Columbia (Col-0) plants grown in soil under normal conditions (Fig. S1A). Eleven strains belonging to three bacterial phyla were isolated (Fig. S1B). We randomly chose three strains from each phylum for subsequent testing: *Bacillus* sp. C3 (Firmicutes), *Micrococcus* sp. C5 (Actinobacteria), and *Blastomonas* sp. C10 (Alphaproteobacteria) (Fig. S1B). These three bacteria were injected separately into the leaves of 4-week-old soil-grown Col-0 plants. None of them caused disease-like symptoms in leaves, even at high bacterial population densities (10^8^ CFU ml^−1^) (Fig. S1C). In an agar plate system [43], the three bacteria also failed to cause noticeable disease symptoms in Col-0 leaves (Fig. S1D). These results indicate that the three selected bacteria are non-pathogenic to the leaves of Col-0 plants.

**Figure 1:**
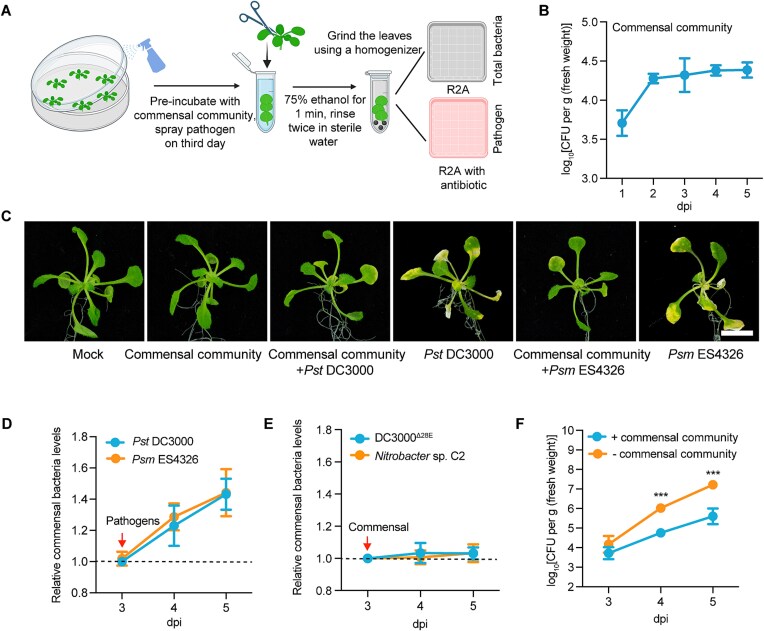
The commensal bacterial population in Col-0 leaves increases during early pathogen invasion. (**A**) Schematic diagram showing how the effect of pathogens on the commensal community in leaves was evaluated. Two-week-old plants were preincubated with the commensal community, and pathogens (10^6^ CFU mL^−1^) were sprayed onto the plants on the third day of culture. The leaves were surface-sterilized and the bacterial population was counted; the population of commensal bacteria was determined by subtracting the number of pathogenic bacteria on selective medium from the total bacterial count; (**B**) population dynamics of the commensal community in Col-0 leaves sprayed with 10^6^ CFU mL^−1^ of commensal bacteria; (**C**) representative images of Col-0 plants sprayed with bacterial suspension at a concentration of 10^6^ CFU mL^−1^. The plants were pre-sprayed with commensal community, and pathogens were spayed after 3 days. Photographs were taken 5 days after spaying with pathogens. Bar, 0.5 cm; (**D**) Changes in the commensal bacterial population in Col-0 following invasion by the pathogen *Pst* DC3000 or *Psm* ES4326. The leaves were incubated with commensal community for 3 days, and pathogens (10^6^ CFU mL^−1^) were added on the third day. Relative commensal bacteria levels were calculated by dividing the population of the commensal community incubated with pathogens by that of the community not incubated with pathogens; (**E**) the effects of the non-pathogens DC3000^*Δ*28E^ and *Nitrobacter* sp. C2 (10^6^ CFU mL^−1^) on the population of endophytic bacteria; plants were treated with DC3000^*Δ*28E^ of *Nitrobacter* sp. C2 after 3 days of culture; (**F**) the population dynamics of *Pst* DC3000 in Col-0 leaves in the presence and absence of commensal bacteria. Experiments were performed three times. Data were analyzed by a two-sided Student’s t test, *** indicates a significant difference (*P* < 0.001) at the indicated time point. Dpi, days post-inoculation.

To investigate how pre-existing endophytic bacteria in leaves respond to pathogen invasion, we formed a commensal community using the three selected bacteria and examined the dynamic changes between the community and pathogenic bacteria (Fig. 1A; see Methods). In Col-0 plants, the population of each species within the commensal community stabilized within 2 days post-inoculation (Fig. S1E). Despite initially exhibiting rapid proliferation, the population density of *Bacillus* sp. C3 also stabilized after 2 days (Fig. S1E). After an initial increase, the commensal community in Col-0 leaves reached an equilibrium (Fig. 1B). The commensal community does not cause any chlorosis phenotypes, it can alleviate chlorosis symptoms caused by the pathogens *Pst* DC3000 and *Psm* ES4326 [28] (Fig. 1C). Similarly, the population of commensal bacteria in the leaves became unstable and rapidly proliferated within 24 h after the pathogens (*Pst* DC3000 and *Psm* ES4326) were introduced into the Col-0 leaves (Fig. 1D). When we treated the leaves with the non-pathogenic bacterium DC3000^*Δ*28E^ or the isolated non-pathogenic bacterium *Nitrobacter* sp. C2, the growth of the commensal community was not significantly affected (Fig. 1E).

The pathogen *Pst* DC3000 exhibited exponential growth, multiplying ~100-fold daily within the leaf (Fig. 1F). However, this rapid proliferation was attenuated when the leaves were pre-conditioned by 3 days of co-culture with the commensal community, indicating that the microbial community, including its rapid proliferation after pathogen invasion, had a profound effect on pathogen dynamics (Fig. 1F).

Collectively, these results suggest that in Col-0 leaves, the population of commensal bacteria is maintained at a stable level under normal conditions. However, during early pathogen (e.g. *Pst* DC3000) invasion (<24 h), pre-existing commensal bacteria exhibit rapid increases in population density.

### Commensal bacteria response to *Pst* DC3000 invasion specifically via the receptor kinase FER

**Figure 2:**
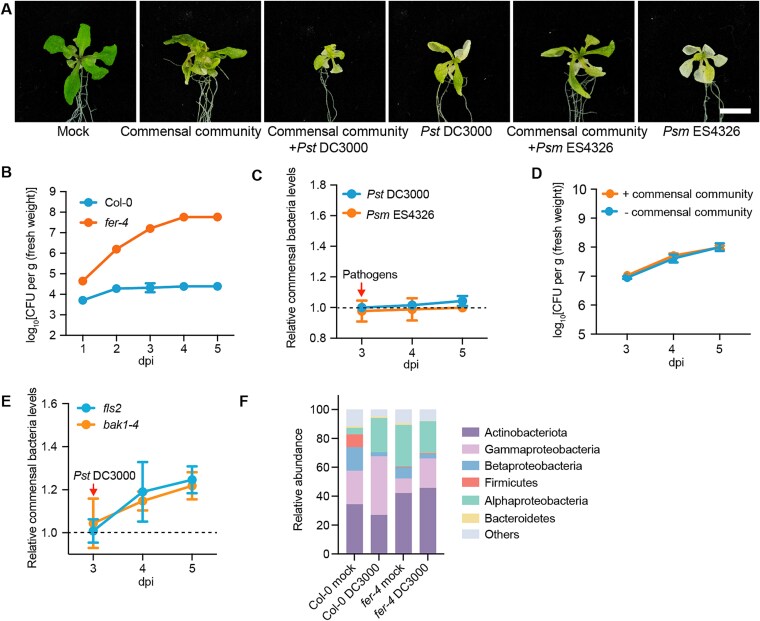
**Commensal bacteria in *fer-4* leaves do not respond to invasion by *Pst* DC3000.** (**A**) Representative images of *fer-4* plants sprayed with bacterial suspension at a concentration of 10^6^ CFU mL^−1^. The plants were pre-sprayed with commensal community, and pathogens were spayed after 3 days. Photographs were taken 5 days after spaying with pathogens. Bar, 0.5 cm; (**B**) the population of the commensal community in *fer-4* leaves sprayed with bacteria at a density of 10^6^ CFU mL^−1^, the data for Col-0 are the same as in Fig. 1B; (**C**) changes in the commensal bacterial population in *fer-4* leaves following invasion by the pathogen *Pst* DC3000 or *Psm* ES4326. The leaves were incubated with the commensal community for the first 3 days, and pathogens (10^6^ CFU mL^−1^) were added on the third day. Relative commensal bacteria levels were calculated by dividing the population of the commensal community incubated with pathogens by that of the community not incubated with pathogens; (**D**) the population of *Pst* DC3000 in *fer-4* leaves with or without commensal bacteria; (**E**) changes in the commensal bacterial population in *fls2* and *bak1–4* leaves following invasion by the pathogen *Pst* DC3000; (**F**) relative abundance of endophytic leaf bacteria at the phylum level and class level for Proteobacteria. Experiments were repeated three times. Dpi, days post-inoculation.

Wild-type Arabidopsis recruit commensal bacteria in response to early pathogen invasion, but the underlying mechanism is unknown. Several plant proteins regulate bacterial community structure in plants, such as RBOHD [19], CERK1 [44], and FER [19, 45]. Loss-of-function mutations of FER receptor kinases significantly influence the bacterial community in the phyllosphere [19], and FER also affects plant defense against pathogens [46–49]. We therefore reasoned that FER might play a role in the mechanism by which commensal bacteria respond to the pathogen *Pst* DC3000. To test this hypothesis, we examined how the commensal community responds to pathogens in *fer-4* (*FER* mutant) leaves. The commensal community cannot alleviate the chlorosis symptoms caused by pathogens (*Pst* DC3000 and *Psm* ES4326) in *fer-4* plants (Fig. 2A) as it does in Col-0 plants. In fact, as the commensal community grows, it can itself cause chlorosis symptoms in *fer-4* (Fig. 2A). We then examined the population of commensal bacteria in *fer-4* leaves. The population of commensal bacteria increased rapidly in *fer-4* leaves sprayed with 10^6^ CFU ml^−1^ of this bacterial community, reaching an equilibrium after 5 days (Fig. 2B). The bacterial population was ~10 000 times that in Col-0 (Fig. 2B and Fig. S2). When the pathogens *Pst* DC3000 and *Psm* ES4326 were introduced into *fer-4* leaves, the commensal community showed no significant responses to the pathogens, as the bacterial community did not exhibit notable changes in population in the presence or absence of the pathogens (Fig. 2C). Similarly, the growth of *Pst* DC3000 in *fer-4* leaves showed no significant changes in the presence or absence of the commensal community (Fig. 2D). These results suggest that *fer-4* plants have lost the ability to recruit pre-existing commensal bacteria in response to pathogen invasion, leading to a faster growth rate of pathogens in *fer-4* than in wild-type Col-0, which is consistent with previous findings [50].

To determine whether the changes in the leaf endophytic bacterial community in *fer-4* are specifically due to *FER* mutation–induced changes in immunity, we examined the variations in the leaf bacterial community in two additional immunity-related mutants: *fls2* and *bak1–4* [51]. The commensal community proliferated rapidly in *fls2* and *bak1–4* leaves, similar to Col-0 (Fig. 2E). These results suggest that the response of the leaf commensal bacteria to *Pst* DC3000 specifically relies on FER.

Based on the above results, we suggest that the pathogens response of endophytic commensal bacteria is diminished in *fer-4* leaves, resulting in increased bacterial presence within leaves. Perhaps under soil-grown conditions, a greater number of bacteria, including harmful ones, enter the leaves of *fer-4* plants. This could potentially cause dysbiosis in the leaves. To test this hypothesis, we measured the population of leaf endophytic bacteria in soil-grown *fer-4*. The population of endophytic bacteria in leaves was higher in *fer-4* than in Col-0 (Fig. S3A). However, there was little difference in the total leaf bacterial population between Col-0 and *fer-4* plants (Fig. S3A). Analysis of the Shannon index also showed that the endophytic leaf community in *fer-4* plants was substantially more diverse and richer than that of Col-0 (Fig. S3B). We also isolated the endophytic bacteria from *fer-4* leaves (Fig. S4A) and injected this bacterial community into the leaves of 4-week-old Col-0 plants. The presence of the endophytic community from *fer-4* (SynCom*^fer-4^*) resulted in prominent necrosis and chlorosis in Col-0 leaves under both soil-grown (Fig. S4B) and gnotobiotic conditions (Fig. S4C). The SynCom*^fer-4^* population was larger than that of SynCom^Col-0^ (Fig. S4D), supporting the hypothesis that *fer-4* leaves experience dysbiosis and are enriched with tissue-damaging bacteria.

To explore the response of the endophytic microbial community to pathogen invasion under normal growth conditions, we conducted 16S rRNA gene sequencing of the leaf bacterial communities of Col-0 and *fer-4* plants after injecting them with low densities of *Pst* DC3000 (10^5^ CFU ml^−1^). The invasion of *Pst* DC3000 caused significant changes to the endophytic microbial community in Col-0 leaves, which were characterized by a marked increase in Alphaproteobacteria and Gammaproteobacteria levels, along with a decrease in Firmicutes and Betaproteobacteria levels (Fig. 2F). By contrast, the disturbance to the microbial community in *fer-4* leaves was relatively minor (Fig. 2F). These results support the notion that FER is required for the response of commensal bacteria to *Pst* DC3000 invasion.

### AvrPto secreted by *Pst* DC3000 interacts with FER and inhibits its kinase activity

**Figure 3:**
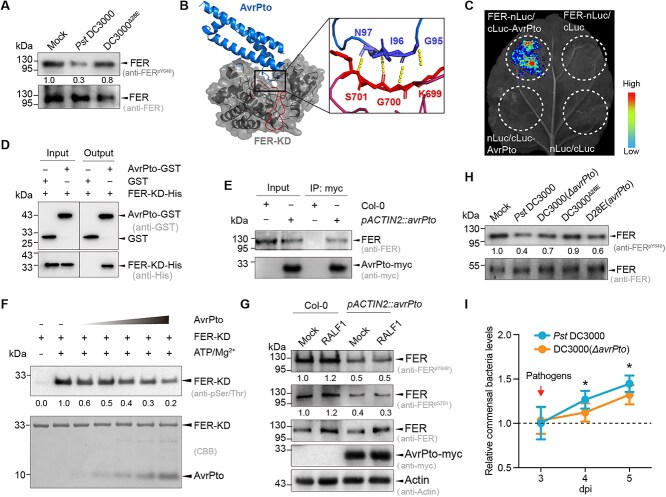
**AvrPto interacts with FER and inhibits its kinase activity.** (**A**) Analysis of the effects of *Pst* DC3000 and DC3000^*Δ*28E^ on the phosphorylation of FER in Col-0 leaves; ﻿(**B**) the structure of the AvrPto–FER complex predicted by ColabFold based on the crystal structures of AvrPto (2QKW) and FER-KD (7XDV); (**C**) AvrPto interacts with FER in a luciferase complementation assay; (**D**) GST pull-down assay showing that AvrPto interacts with FER-KD. Recombinant AvrPto-GST was co-incubated with his-tag FER-KD (FER-KD-His), and the protein complex was affinity-purified with glutathione-conjugated agarose beads; (**E**) AvrPto interacts with FER in a co-immunoprecipitation (CoIP) assay. Proteins were extracted from 10-day-old Col-0 and *pACTIN2::avrPto* transgenic Arabidopsis 2 h post-inoculation for CoIP with myc beads. FER and AvrPto-myc proteins were detected by immunoblotting with anti-FER and anti-myc antibody, respectively; (**F**) FER-KD autophosphorylation is inhibited by AvrPto in vitro. FER-KD (1 mM) and AvrPto were incubated with 10 mM ATP and 5 mM MgCl_2_ at 25°C for 30 min; (**G**) FER-KD phosphorylation is inhibited by AvrPto in vivo. The phosphorylation of Y648 and S701 of FER was detected in 7-day-old *pACTIN2::avrPto* seedlings. As a positive control, 200 nM RALF1 was added for inducing phosphorylation of FER. The results are representative of three independent experiments; (**H**) FER is phosphorylated following treatment with DC3000(*ΔavrPto*) and D28E(*avrPto*); (**I**) the commensal bacterial population in Col-0 leaves changes after *Pst* DC3000 and DC3000(*ΔavrPto*) treatment. The leaves were incubated with commensal bacteria for the first 3 days, and *Pst* DC3000 or DC3000(*ΔavrPto*) (10^6^ CFU mL^−1^) was added on the third day. Relative commensal bacteria levels were calculated by dividing the population of the commensal community incubated with *Pst* DC3000 or DC3000(*ΔavrPto*) by that of the community not incubated with those two strains. Dpi, days post-inoculation. Experiments were performed three times. Data were analyzed by a two-sided Student’s t test, * indicates significant difference (*P* < 0.05) at the indicated time point.

The kinase activity of FER is indispensable for its functions in controlling several processes, including root growth, aerial development, jasmonate signaling, and vacuolar expansion [52, 53]. We measured the phosphorylation of FER of Col-0 leaves by immunoblotting using anti-FER^pY648^ antibody [54] following incubation with the pathogen *Pst* DC3000 and the non-pathogen DC3000^*Δ*28E^. Under normal conditions, the phosphorylation of FER was readily detectable (Fig. 3A). Leaf infiltration with *Pst* DC3000 resulted in a significant reduction in FER phosphorylation after 24 h (Fig. 3A). The FER phosphorylation level in Col-0 leaves infiltrated with DC3000^*Δ*28E^ was substantially restored compared to leaves infiltrated with wild-type *Pst* DC3000 (Fig. 3A). These observations suggest that the pathogen *Pst* DC3000 inhibits the kinase activity of FER, dampening the response of leaf commensal bacteria to *Pst* DC3000 invasion.

The absence of 28 effectors in DC3000^Δ28E^, compared to *Pst* DC3000, accounts for the observed variation in FER kinase activity. *Pst* DC3000 effectors may specifically target FER, thereby modulating its phosphorylation. To identify FER-interacting effectors, we used ColabFold [37] to assess the predicted interactions between FER and the 28 effector proteins of *Pst* DC3000. Twelve effector–FER complexes exhibited pLDDT scores >70, indicating a high probability of interaction (Fig. S5A). In a yeast two-hybrid assay to verify the interactions between these effectors and FER, AvrPto showed a strong interaction with FER (Fig. S5B). We employed ColabFold to more precisely predict the significant interaction between AvrPto [55] and FER-KD [41] (residues 518–816) based on the crystal structures of the proteins. The results indicated that AvrPto showed a clear binding affinity to FER-KD (Fig. 3B).

The interaction between AvrPto and FER was further validated using several complementary approaches. Luciferase complementation assays in *N. benthamiana* confirmed that AvrPto interacts with FER (Fig. 3C). Additionally, glutathione *S*-transferase (GST) pull-down assays confirmed the interaction between AvrPto-GST and His-tagged FER-KD (Fig. 3D). Finally, in CoIP assays using*pACTIN2::avrPto* transgenic Arabidopsis, AvrPto-myc co-immunoprecipitated with FER (Fig. 3E). Collectively, these results demonstrate that AvrPto and FER physically interact.

The effector AvrPto has been shown to inhibit the phosphorylation of several proteins, including the serine/threonine kinase Pto [55] and the receptor kinases FLS2 and EFR [17]. The crystal structures of Pto and FER-KD are highly similar, with a root mean square deviation of 0.547 Å (Fig. S5C). ColabFold predicted that AvrPto binds to the activation domain of FER-KD [41], with three residues forming hydrogen bonds (Fig. 3B). This structural similarity suggests that AvrPto may influence FER phosphorylation. To investigate the effect of AvrPto on FER phosphorylation, we examined the phosphorylation status of FER-KD, the core kinase domain of FER that interacts with AvrPto. The autophosphorylation of FER-KD decreased with increasing concentrations of AvrPto (Fig. 3F). We then examined the *in vivo* phosphorylation of FER using anti-FER^pY648^ and anti-FER^pS701^ antibodies. The phosphorylation of FER was inhibited in*pACTIN2::avrPto*, even when using the FER activator RALF1, a peptide known to trigger the phosphorylation of FER [56] (Fig. 3G). These results demonstrate that AvrPto inhibits the kinase activity of FER.

To investigate the functional significance of AvrPto-mediated inhibition of FER, we injected the strain DC3000(*ΔavrPto*), with an *avrPto* gene knockout, into Col-0 leaves. Compared to wild-type *Pst* DC3000, leaves treated with DC3000(*ΔavrPto*) exhibited a significantly higher level of FER phosphorylation, indicating that *Pst* DC3000 employs AvrPto to inhibit FER activation within the leaves (Fig. 3H). We quantified the populations of the commensal community in Col-0 leaves following treatment with DC3000(*ΔavrPto*) in Col-0 leaves. Compared to the wild-type *Pst* DC3000, the population of commensal bacteria was significantly decreased when the leaves were sprayed with DC3000(*ΔavrPto*) (Fig. 3I). These results suggest that the effector AvrPto targets FER, inhibiting its kinase activity, and that AvrPto is necessary for commensal bacteria to response to *Pst* DC3000 invasion.

### Detection of pathogen invasion depends on FER kinase activity

**Figure 4:**
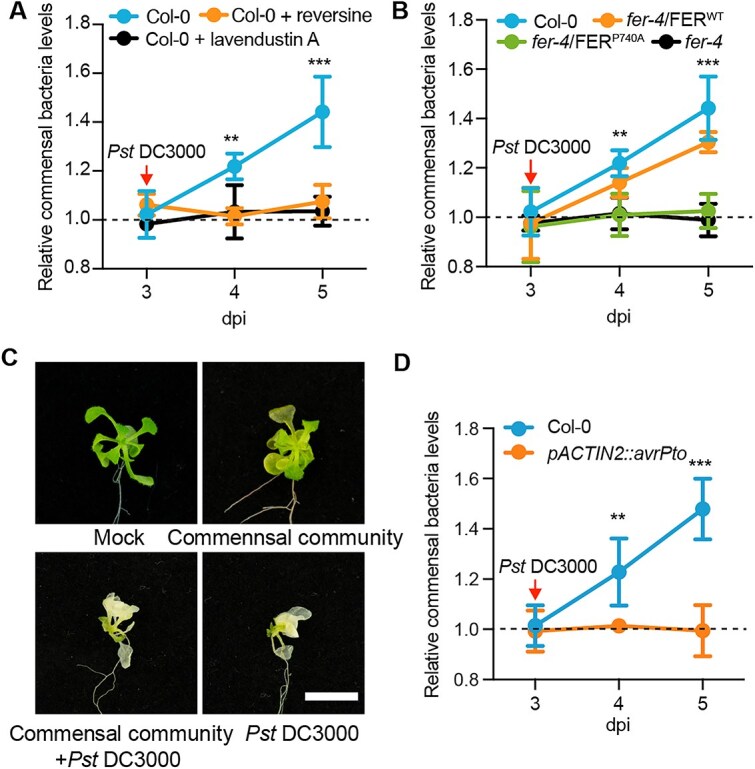
**The response of commensal bacteria to *Pst* DC3000 invasion is dependent on the kinase activity of FER.** (**A**) Changes in the commensal bacterial population in Col-0 leaves in response to invasion by *Pst* DC3000 after treatment with 5 μM FER kinase inhibitors (reversine and lavendustin A). Relative commensal bacteria levels were calculated by dividing the population of the commensal community incubated with pathogens by that of the community not incubated with pathogens; (**B**) Changes in the commensal bacterial populations in Col-0, *fer-4*/FER^WT^, *fer-4*/FER^P740A^, and *fer-4* leaves in response to invasion by *Pst* DC3000; (**C**) representative images of *pACTIN2::avrPto* plants were sprayed with bacterial suspension at a concentration of 10^6^ CFU mL^−1^. The plants were pre-sprayed with commensal bacteria, and pathogens were sprayed after 3 days. Photographs were taken 5 days after spaying with pathogens. Bar, 0.5 cm; (**D**) Changes in the commensal bacterial population in Col-0 and *pACTIN2::avrPto* leaves in response to invasion by *Pst* DC3000. Experiments were performed three times. Asterisks indicate significant differences at the indicated time points, as analyzed by a two-sided Student’s t test, where **, *P* < 0.01; ***, *P* < 0.001. dpi, days post-inoculation.

Our results indicate that AvrPto inhibits the kinase activity of FER, thereby affecting the response of leaf commensal bacteria to *Pst* DC3000 infection. To further validate this conclusion, we utilized two FER kinase inhibitors, reversine and lavendustin A, which are ATP-competitive inhibitors that target the ATP binding pocket of FER [54]. By treatment with these inhibitors, the endophytic commensal bacteria exhibited a reduced response to invasion by *Pst* DC3000 compared to the control (Fig. 4A).

We examined *fer-4*/FER^P740A^, an Arabidopsis FER mutant in the *fer-4* background with a mutation at the P740 site within the dimerization interface of FER. This mutation inhibits the allosteric activation of FER and significantly diminishes its kinase activity [25]. Consistently, the commensal bacteria in *fer-4*/FER^P740A^ plants showed no response to *Pst* DC3000, paralleling the response in *fer-4* (Fig. 4B). By contrast, the complementary mutant *fer-4*/FER^WT^ exhibited behavior consistent with that of wild-type Col-0 (Fig. 4B). Collectively, these findings suggest that the kinase activity of FER plays a critical role in regulating the response of commensal bacteria in leaves to invasion by the pathogen *Pst* DC3000.

To further confirm the roles of the AvrPto–FER module, we assessed the response of commensal bacteria in *pACTIN2::avrPto* transgenic plants, which continuously overexpress AvrPto, to *Pst* DC3000. The commensal bacteria caused the obvious chlorosis symptoms in *pACTIN::avrpto* plants and were unable to reduce the disease symptoms caused by the pathogen *Pst* DC3000 (Fig. 4C). We measured the changes of endophytes within the leaves, and the commensal bacteria showed no significant response to the pathogen in *pACTIN2::avrPto* plants (Fig. 4D). These findings indicate that the AvrPto-mediated inhibition of FER kinase activity compromises the plant response to *Pst* DC3000 infection.

### Commensal bacteria respond to *Pst* DC3000 via FER/RIPK–RBOHD module- involved PTI pathway

**Figure 5:**
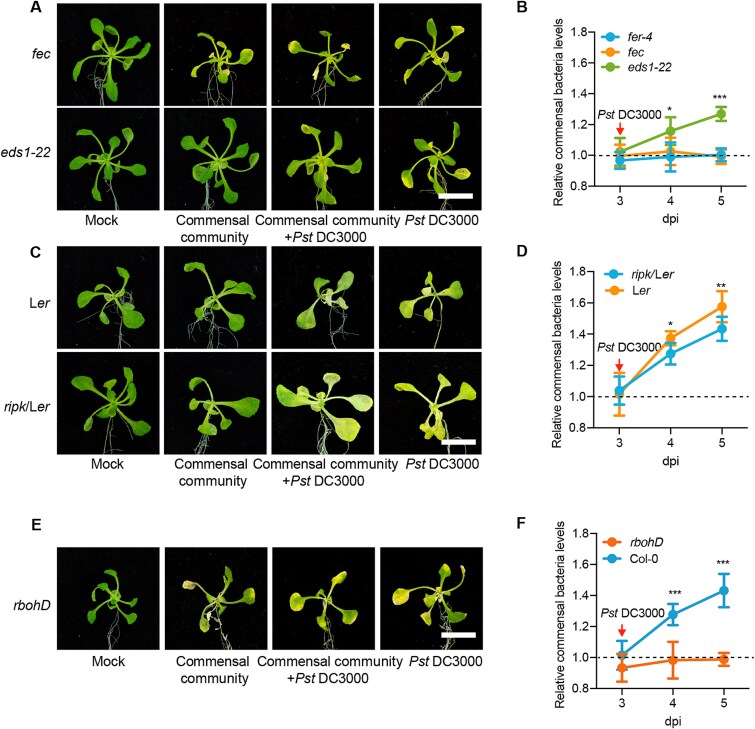
**FER affects the response of commensal bacteria to *Pst* DC3000 invasion via RIPK–RBOHD and PTI.** (**A**) Representative images of *fec* and *eds1-22* plants sprayed with bacterial suspension at a concentration of 10^6^ CFU mL^−1^. The plants were pre-sprayed with commensal community, and pathogens were spayed after 3 days. Photographs were taken 5 days after spaying with pathogens. Bar, 0.5 cm; (**B**) Changes in the commensal bacterial population in *fer-4*, *fec*, and *eds1-22* leaves in response to invasion by *Pst* DC3000. Relative commensal bacteria levels were calculated by dividing the population of the commensal community incubated with pathogens by that of the community not incubated with pathogens; (**C**) Representative images of L*er* and *ripk/*L*er* plants sprayed with bacterial suspension at a concentration of 10^6^ CFU mL^−1^. The plants were pre-sprayed with commensal community, and pathogens were spayed after 3 days. Photographs were taken 5 days after spaying with pathogens. Bar, 0.5 cm; (**D**) Changes in the commensal bacterial population in *ripk*/L*er* and L*er* leaves in response to invasion by *Pst* DC3000; (**E**) Representative images of *rbohD* plants sprayed with bacterial suspension at a concentration of 10^6^ CFU mL^−1^. The plants were pre-sprayed with commensal community, and pathogens were spayed after 3 days. Photographs were taken 5 days after spaying with pathogens. Bar, 0.5 cm; (**F**) Changes in the commensal bacterial population in *rbohD* and Col-0 leaves in response to invasion by *Pst* DC3000. The plants were sprayed with the commensal bacteria community (10^6^ CFU mL^−1^) on the first day and treated with *Pst* DC3000 (10^6^ CFU mL^−1^) on the third day. Experiments were repeated three times. Asterisks indicate significant differences at the indicated time points, as analyzed by a two-sided Student’s t test, where *, *P* < 0.05; **, *P* < 0.01; ***, *P* < 0.001.

FER is a key component of the immune system in Arabidopsis [52, 57]. To verify the immune mechanism by which FER regulates the response of commensal bacteria to pathogen invasion, we investigated how commensal bacteria respond to *Pst* DC3000 in the PTI-deficient mutant *fls2 efr cerk1* (*fec*) [29] and the ETI-deficient mutant *eds1-22* [24]. The commensal bacteria caused chlorosis in the *fec* mutant, but no chlorosis was observed in the *eds1-22* mutant, which appeared healthy (Fig. 5A). In the *fec* mutant, the commensal community is unable to alleviate the chlorosis caused by *Pst* DC3000, whereas it can do so in the *eds1-22* mutant (Fig. 5A). Consistent with chlorosis, the population of the commensal community rapidly proliferated in response to *Pst* DC3000 invasion in *eds1-22*. By contrast, the commensal bacteria in the *fec* mutant exhibited no significant reaction to *Pst* DC3000 invasion, mirroring the response observed in *fer-4* (Fig. 5B). These findings suggest that commensal bacteria respond to *Pst* DC3000 specifically via the PTI pathway, which includes FER.

To investigate how the AvrPto–FER module affects PTI responses, we focused on the FER-associated receptor-like cytoplasmic kinase (RLCK) RIPK. RIPK facilitates cross-phosphorylation with FER and transmits RALF signals, which are received by FER [23]. In leaves of an *RIPK* mutant in the Landsberg *erecta* (L*er*) background (*ripk*/L*er*), the ability of the commensal community to respond to *Pst* DC3000 was reduced compared to that in leaves of wild-type L*er* (Fig. 5C and D). In *ripk*, unlike *fer-4*, the response of commensal bacterial to the pathogen was reduced rather than completely lost. We suggest that FER functions with several RLCK partner proteins to control its downstream processes [58].

RIPK functions in the plant immune system by generating reactive oxygen species (ROS) through the phosphorylation of RBOHD [59], which regulates PTI. Based on the above observations, we reasoned that RBOHD might also play a role in regulating the response of commensal bacteria to the presence of pathogen in leaves via the PTI pathway. We therefore examined the commensal bacteria response to the pathogen in the *rbohD* mutant. In this mutant, the commensal bacteria can cause chlorosis and are unable to alleviate the chlorosis caused by *Pst* DC3000 (Fig. 5E). We also measured the changes in the population of commensal bacteria in response to the presence or absence of the pathogen. In the *rbohD* mutant, commensal bacteria did not respond to *Pst* DC3000 invasion (Fig. 5F), indicating that RBOHD is indeed required for this response.

## Discussion

In nature, plants depend on pre-existing commensal microorganisms to maintain their health [6, 60]. However, the intricate interplay among plants, commensal bacteria, and pathogens remains somewhat unclear. In particular, little is known about how plants communicate with their non-pathogenic microbial communities in response to pathogens. To understand how plants fine-tune their immune systems in response to pathogens, it is crucial to explore the complex relationships between plant immunity and the endophytic microbiota. Such an understanding could reveal plants optimize their immune responses to foster the growth of beneficial or commensal microbes while defending against harmful pathogens. In this study, we analyzed the roles of the plant receptor kinase FER in regulating the growth of leaf endophytic commensal bacteria in response to early pathogen invasion. From the perspective of plant gene analysis, it dissects how the gene specifically regulates the response of commensal bacteria to pathogens.

Although multiple studies have investigated how plants sense pathogen invasion, they mainly focused on PRRs on the cell surface, which recognize pathogen- and damage-associated molecular patterns during initial invasion [61], while less is known about the response of endophytic commensal bacteria in plants to pathogen entry, especially during the early recognition stage. The *fer* mutant, which exhibits pleiotropic growth defects, was reported to have a close relationship with microorganisms. For example, the *fer* mutant was found to be more resistant to powdery mildew and *Fusarium oxysporium* infection [46, 47], but more susceptible to the bacterial pathogen *Pst* DC3000 [49, 50]. Furthermore, the *fer* mutant had the most significant influence on the composition of the phyllosphere community compared to several other Arabidopsis immunity mutants [19]. It indicates that the role of FER in regulating plant microorganisms goes beyond our understanding. Our data showed that FER plays a crucial role in influencing the endophytic community, contributing to the maintenance of endophytic bacteria populations within a balanced range (Fig. 2A) and special adjusting the structure and populations of these bacteria in response to the initial invasion of pathogens (Fig. 2F).

In the Arabidopsis phyllosphere, strains belonging to the phyla Proteobacteria and Actinobacteria help protect the plant against infection by *Pst* DC3000 [6]. Our results reveal that the endophytic commensal bacteria rapidly respond to *Pst* DC3000 (in <24 h). Alphaproteobacteria and Gammaproteobacteria positively respond to pathogen invasion, and the abundance of Actinobacteria decreases. These results indicate that Proteobacterial strains provide protection in both the phyllosphere and endosphere of leaves during pathogen invasion. Changes in the endophytic community structure in response to pathogen invasion have also been observed in other plants. The pathogen *Xanthomonas oryzae* pv. *oryzae* induces a decrease in the alpha diversity of fungal communities while increasing the bacterial communities within the endophytic microbiota of rice leaves [62]. The endophytic leaves of *Paullinia cupana* exhibited higher microbial diversity following infection by the pathogen *Colletotrichum* spp. [63]. Similarly, in the human gut, larger populations of beneficial bacteria provide a more effective defense against pathogens [64]. Therefore, artificially manipulating the changes in endophyte community structure may be an effective way to prevent pathogens.

The secretion of effectors by *Pst* DC3000 to suppress plant immunity is an early event in infection [3]. We determined that FER kinase activity was inhibited by the effector AvrPto. Subsequently, the population of commensal bacteria increased in response to *Pst* DC3000 invasion due to the suppression of FER-mediated immunity, leading to slower early-stage proliferation of *Pst* DC3000 in leaves. Therefore, we discovered another function of FER: sensing *Pst* DC3000 invasion through AvrPto and paving the way for proliferation of commensal bacteria in response. Our findings indicate that the effector AvrPto plays a dual role: its primary function is to suppress plant immunity to facilitate pathogen growth, yet it also creates opportunities for the proliferation of commensal bacteria. The recognition of AvrPto by plants and its promotion of commensal bacterial growth are specifically dependent on the kinase activity of FER. This pathway is distinct from the changes in immunity induced by mutations in *FLS2* and *BAK1* (Fig. 2C). The FLS2–BAK1 complex assembles upon induction by the flagellin fragment flg22, which activates the plant innate immune response [65]. In Arabidopsis roots, FER maintains the structure of the rhizosphere microbiome by regulating ROP-RBOHs-mediated basal ROS production [19]. In this study, we further confirm that FER modulates RBOHD via RIPK to influence plant PTI immunity, thereby providing commensal bacteria with opportunity to respond to pathogens.

**Figure 6:**
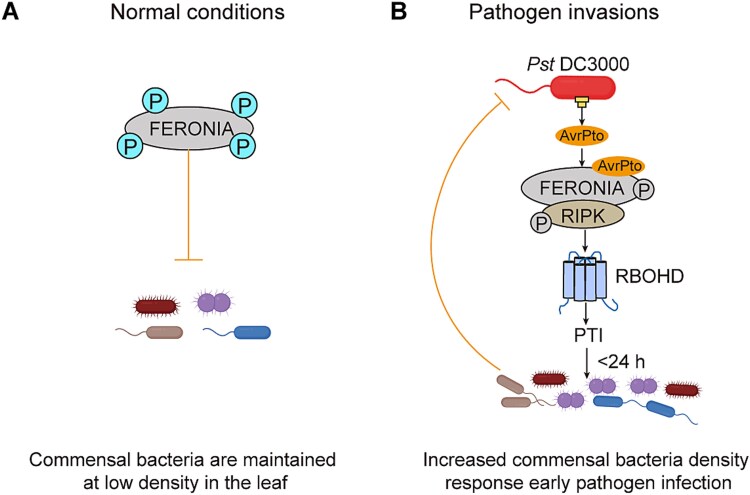
**Mechanistic model for the role of FER in regulating commensal bacteria response to pathogens. (A)** Under normal conditions, FER plays a vital role in controlling the excessive growth of endophytic bacteria in the leaf and maintaining a balanced microbial population. **(B)** When pathogens invade, they inhibit the kinase activity of FER by releasing the effector AvrPto, decreasing the participation of FER/RIPK–RBOHD in the regulation of PTI. The increased population of commensal bacteria effect early pathogen proliferation.

In conclusion, our study found commensal bacteria specifically relies on the AvrPto-FER-RIPK–RBOHD module to increase their abundance in response to pathogen invasion. Our findings suggest strategies to protect plants from pathogens by modulating receptor kinase activity (Fig. 6). It offers an effective means for the early prevention and control of crop diseases, thereby minimizing agricultural losses and environmental impact associated with post-outbreak treatments.

## Supplementary Material

R2-Supplementary_Figures_wraf145

R2-Supplementary_Table_wraf145

## Data Availability

Raw Illumina data for 16S rRNA gene sequences data of total and endophytic bacterial community have been deposited into the Genome Sequence Archive [66] in National Genomics Data Center (https://ngdc.cncb.ac.cn/gsa) [67], China National Center for Bioinformation/Beijing Institute of Genomics, Chinese Academy of Sciences, under BioProject PRJCA038564, with accession numbers CRA025116. The full 16S rRNA gene sequences of endophytic strains isolated from Arabidopsis leaves have been deposited in the GenBase [68] in National Genomics Data Center. The accession numbers for strains from the *fer-4* mutant range from C_AA108190.1 to C_AA108200.1, while those for strains from Col-0 range from C_AA108201.1 to C_AA108211.1. These sequences are publicly accessible at https://ngdc.cncb.ac.cn/genbase.
