# Supplementary material for: Endophytic commensal bacteria capitalize on the AvrPto-FER pathway to enhance proliferation during early stages of pathogen invasion
Source: ISME J. 2025 Jul 9;19(1):wraf145. doi: 10.1093/ismejo/wraf145 (PMC12341878; doi:10.1093/ismejo/wraf145)
Supplement: R2-Supplementary_Figures_wraf145 [file r2-supplementary_figures_wraf145.pdf]

Endophytic commensal bacteria capitalize on the AvrPto-FER pathway to enhance proliferation during early stages of pathogen invasion

Yi Zhang[1, 2, 3], Dan Hu[3], Hong-xia Sun[1, 2], Jia Chen[1, 2], Jia-hao Yang[1, 2], Xin-mei Li[1, 2], Xiu-shan Li[1, 2], Yan Chen[1, 2], Feng Yu[1, 2*]

[1] State Key Laboratory of Chemo/Biosensing and Chemometrics, College of Biology, Longping Agricultural College, Hunan University, Changsha 410082, China

[2] Yuelushan Laboratory, Changsha 410128, China

[3] Hunan Institute of Microbiology, Changsha, 410009, China

*Correspondence: feng_yu@hnu.edu.cn (F. Yu); Tel: +86 731 8882 3646

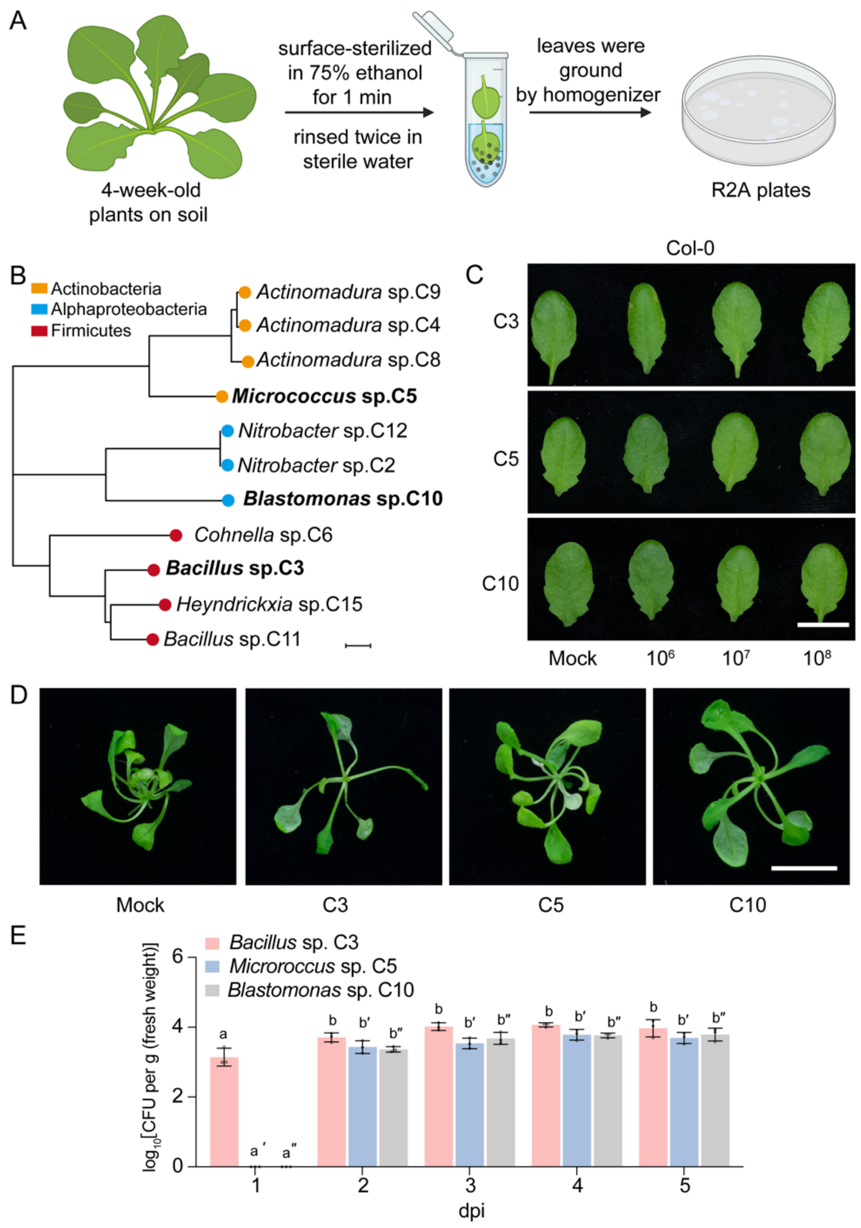

Figure S1. Isolation of bacterial colonies and functional identification of strains. (**A**) Schematic diagram of the isolation of endophytic bacteria from leaves of 4-week-old soil-grown Arabidopsis; (**B**) Phylogenetic relationship of the srains isolated from Col-0 leaves using full-length 16S rRNA gene sequences. The phylogenetic tree was constructed by the maximum-likelihood method using MEGA-X with 1000 replicates, the scale = 0.01; (**C**) Appearance of the leaves of soil-grown 4-week-old Col-0 plants infiltrated with Mock (H_2O) or *Bacillus* sp. C3, *Micrococcus* sp. C5, and *Blastomonas* sp. C10 solution at 10^6, 10^7, and 10^8 CFU mL^{-1}. Images show leaves from bacteria-injected plants at 3 days post-inoculation (dpi). Bar, 1 cm; (**D**) Images of Col-0 plants on plate sprayed (10^6 CFU mL^{-1} bacteria) with *Bacillus* sp. C3, *Micrococcus* sp. C5, and *Blastomonas* sp. C10 at 5 dpi. Bar = 1 cm; (**E**) Endophytic bacterial populations in (D). Data are mean ± s.d. (n = 3 biological replicates) of one representative experiment (out of three independent experiments) analyzed by two-way ANOVA with Tukey's HSD; different letters indicate significant differences ($P < 0.05$).

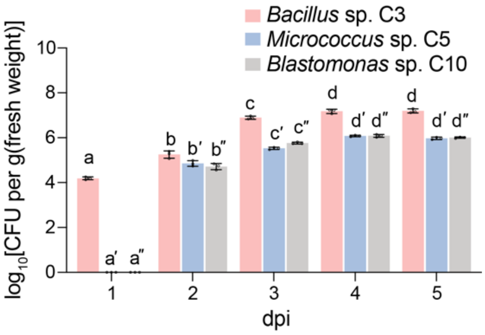

Figure S2 Growth of commensal bacteria in *fer-4* leaves. Endophytic bacterial populations in leaves infiltrated with 10^6 CFU mL^{-1} bacteria. Data are mean ± s.d. (n = 3 biological replicates) of one representative experiment (out of three independent experiments) analyzed by two-way ANOVA with Tukey's HSD; different letters indicate significant differences ($P < 0.05$).

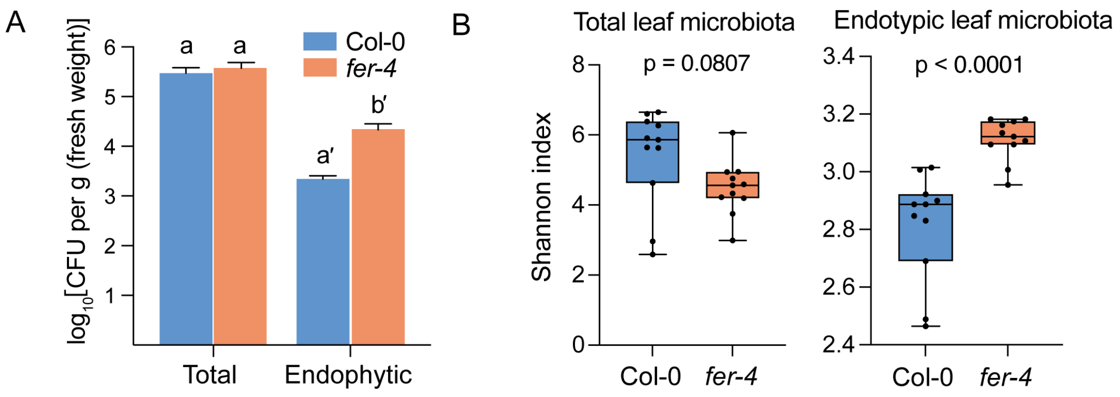

Figure S3 The endophytic bacterial community in *fer-4* leaves exhibits dysbiosis. (**A**) Microbiota population in the leaves of 4-week-old soil-grown Col-0 and *fer-4* plants, data are mean ± s.d. It was analyzed by two-way ANOVA with Tukey's HSD; different letters indicate significant differences ($P < 0.05$); (**B**) Shannon index was obtained from 16S rRNA gene sequence profiles of total bacteria in soil-grown Col-0 and *fer-4* plants, data statistical analysis was performed using a two-sided Student's t test.

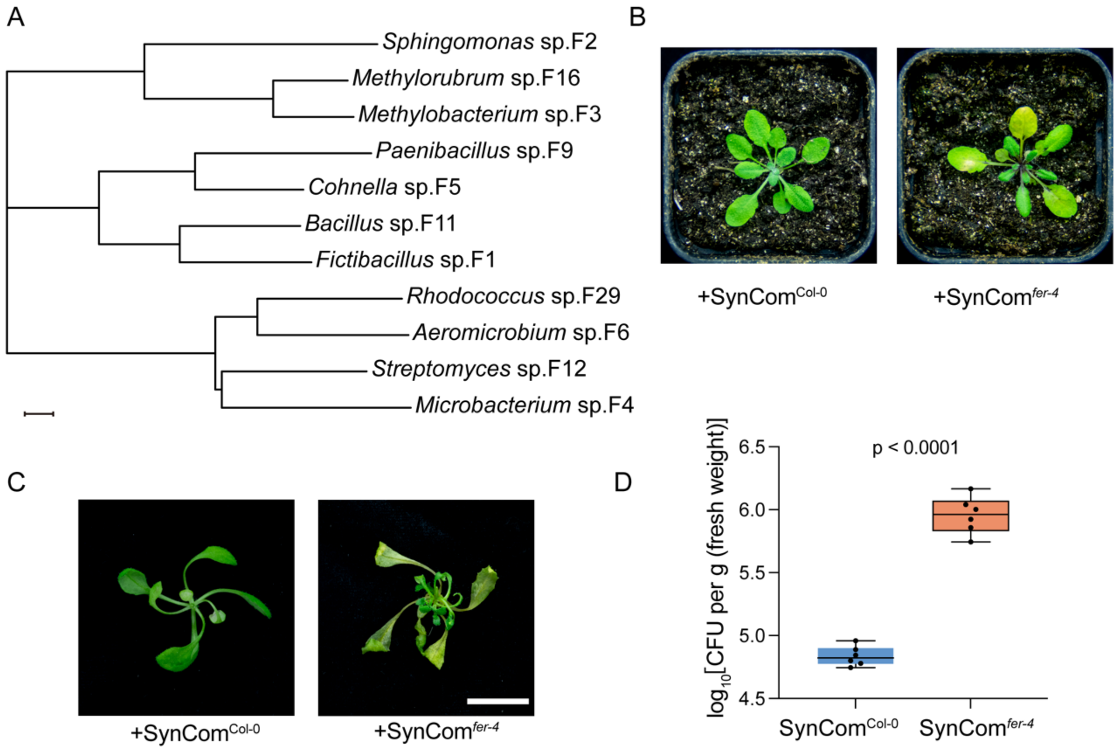

Figure S4 The endophytic bacteria in *fer-4* cause disease symptoms in Col-0 plants. (**A**) Endophytic bacteria isolated from the leaves of 4-week-old soil-grown *fer-4* plants. Phylogenetic relationships of the isolated bacteria were analyzed based on full-length 16S rRNA gene sequences using MEGA-X with 1000 replicates by the maximum-likelihood method, the scale = 0.01; (**B**) Col-0 plants were infiltrated with 10^6 CFU mL^{-1} $SynCom^{Col\text{-}0}$ or $SynCom^{fer\text{-}4}$. The appearance of the plants and the bacterial population was recorded 5 days after infiltration; (**C**) Images of 3-week-old axenic Col-0 plants with $SynCom^{Col\text{-}0}$ and $SynCom^{fer\text{-}4}$ (10^6 CFU mL^{-1} bacteria) in the agar plate system. Bar, 1 cm; (**D**) Bacterial populations recorded 3 days after sprayed, statistical analysis was performed using a two-sided Student's t test.

A

Complex	pLDDT	pTM + ipTM
hopG1-FER	71.9	0.935
hopF2-FER	71.7	1.007
hopR1-FER	71.2	1.322
hopX1-FER	71.1	1.054
hopB1-FER	70.9	0.693
avrPto-FER	70.7	1.006
hopM1-FER	70.5	0.671
hopA1-FER	70.4	0.615
hopU1-FER	70.2	0.994
avrE-FER	70.2	0.993
hopC1-FER	70.2	0.806
hopH1-FER	70.1	0.752
hopO1-1-FER	69.9	0.819
hopT1-1-FER	69.8	1.049
hopE1-FER	69.4	0.731
hopAF1-FER	69.0	0.773
hopAM1-2-FER	69.0	0.845
hopAM1-1-FER	68.4	0.853
hopV1-FER	67.6	0.638
hopAO1-FER	66.4	0.647
hopK1-FER	65.4	0.662
hopN1-FER	64.8	0.904
avrPtoB-FER	63.6	0.992
hopD1-FER	60.9	0.603
hopAA1-2-FER	59.9	0.709
hopY1-FER	58.9	0.643
hopAA1-1-FER	58.9	0.679
hopI1-FER	54.9	0.604

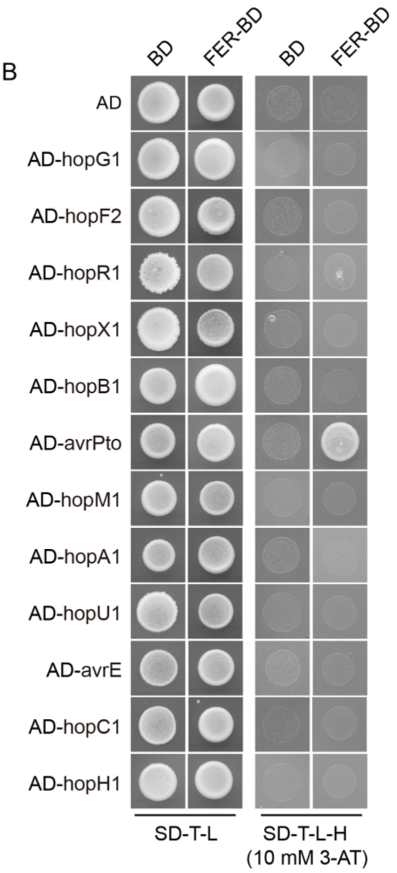

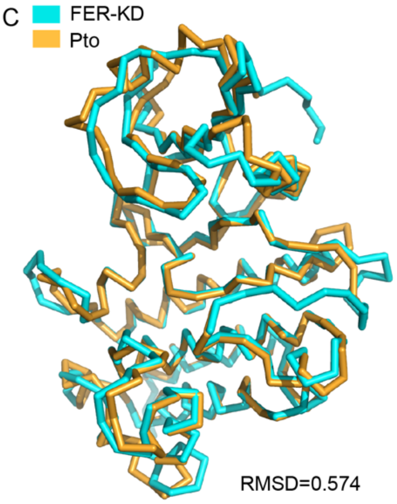

Figure S5 Analysis of the interactions of 28 effector proteins of *Pst* DC3000 with FER. (A) Predicted interactions between effector proteins and FER using ColabFold; the complexes above the red dashed line have a predicted local distance difference test (pLDDT) score > 70; (**B**) Yeast two-hybrid assay to verify the formation of complexes with pLDDT >70; (**C**) Comparison of the structures of Pto (2QKW) and FER-KD (7XDV).
